# 4036 POSITIVE EXPERIENCE OF INFORMED CONSENT UNDERSTANDING AT A METROPOLITAN MULTI-INSTITUTIONS CTSA HUB

**DOI:** 10.1017/cts.2020.137

**Published:** 2020-07-29

**Authors:** Jane Anyasa Otado, Reyneir Magee, John Kwagyan, Sarah Vittone, Debra Ordor, Amy Loveland, MaryAnne Hinkson

**Affiliations:** 1 Georgetown - Howard Universities; 2 Howard University; 3 Georgetown University; 4 MedStar Health Research Institute

## Abstract

OBJECTIVES/GOALS: There is not much known on how to improve informed consent understanding and there are no effective interventions that have been identified to improve understanding rates of information. This study seeks to assess participants understanding of the informed consent. METHODS/STUDY POPULATION: We studied a non-probability sample of 245 participants, 57% female, with age range from 6 to 84, currently enrolled in clinical trials conducted at an urban city, multi CTSA institution. A self-administered questionnaire approved by IRB was utilized. Redcap database was utilized for data entry. The items in the questionnaire reflected understanding of the informed consent (e.g., purpose for the study, participants’ rights, risks, benefits). Participants completed the survey during their first visit to the research centers or on a follow-up visit. Data were collected from July 2018 to November 2019. Data were analyzed descriptively by summary statistics. RESULTS/ANTICIPATED RESULTS: African Americans were 44%, Non-Hispanic Whites were 36%, Hispanic 6%. Others 13%. 52% married, 12% completed High school, 74.8% completed College, 13% less High school. 91% read the form themselves. 99% knew the purpose of the study; 99% knew they could quit the study at any time. While (113) 47% indicated knowledge of the potential risk, only (12)10.6% could not list any associated risk. 98% stated they had information on who to call with questions regarding the study. (204)86% knew of a potential benefit, only (11)5% could not name some study benefit. 38% were unsure/did not know the total number of visits study required of them. 74% knew the duration of the study. DISCUSSION/SIGNIFICANCE OF IMPACT: Extended discussion and more time on one-one by the study teams in this CTSA tend to increase trust. This approach has been reported to be most effective in improving participant understanding of informed consent process and may result in the positive experience.

